# How the environment evokes actions that lead to different goals: the role of object multi-functionality in pavlovian-to-instrumental transfer

**DOI:** 10.1007/s12144-023-04612-2

**Published:** 2023-04-11

**Authors:** Kaiyang Qin, Hans Marien, Ruud Custers, Henk Aarts

**Affiliations:** Department of Psychology, Utrecht University, PO BOX 80140, Utrecht, 3508 TC The Netherlands

**Keywords:** Environmental cues, Goal-directed behavior, Single-functional outcome, Multi-functional outcome, Specific pavlovian-to-instrumental transfer, Outcome value

## Abstract

Research shows that stimuli in the environment can trigger behavior via the activation of goal representations. This process can be tested in the Pavlovian-to-Instrumental Transfer (PIT) paradigm, where stimuli can only affect behavior through the activation of the representation of its desired outcome (i.e., the PIT effect). Previous research has demonstrated that the PIT effect is stronger when the goal is more desirable. While this research only looked at actions that have single outcomes (e.g., obtaining a snack to satisfy appetite), in the present paper, we reason that actions that are instrumental in obtaining outcomes that are desirable in multiple ways (e.g., obtaining a snack to satisfy one’s appetite, giving it to a friend, trading it for money) should produce stronger PIT effects. In two experiments, participants learned to perform left and right key presses to earn a snack, either framed as having a single function or multiple functions. Participants also learned to associate the two differently framed snacks with two cues. In a PIT test, they were required to press the keys as fast as possible upon exposure to the cues (i.e., the PIT effect). We found that cues associated with the multi-functional snack facilitated the actions that earned those snacks before, while cues associated with the single-functional snack did not facilitate such actions. We discuss these findings in the context of research on free choice and personal autonomy and how people appreciate the multi-functional nature of their goal-directed behavior in the environment.

**Supplementary Information:**

The online version contains supplementary material available at 10.1007/s12144-023-04612-2.

## Introduction

Human beings engage in goal-directed behavior. Engaging in goal-directed behavior relies on the ability to represent which actions lead to which desired outcomes or rewards (Dickinson & Balleine, 1994; Prinz, 1997) and to decide which action to execute in order to obtain which outcomes. Although setting a goal and anticipating the desired outcome is often regarded as the starting point for goal-directed action (Gollwitzer, 1990; Locke & Latham, 1990), it has been argued that goal-directed behaviors that are frequently selected in the same context can also be triggered by stimuli in the context that directly activate the mental representation of the goal (Custers & Aarts, 2010). Despite the empirical evidence supporting such environmental control of goal-directed behavior (Weingarten et al., 2016), strict tests of the mediating role of goals in human behavior are scarce.

Such a strict test, though, has been developed in animal research to demonstrate that animal behavior can indeed be mediated by goals. This test has become known as the specific Pavlovian-to-instrumental transfer (PIT) test (Cartoni et al., 2016; Holmes et al., 2010; Mahlberg et al., 2021). The key feature of this paradigm is that it separates the processes of instrumental conditioning (where the animal learns that behavior is instrumental in obtaining an outcome) and Pavlovian conditioning (e.g., where the animal learns that a stimulus is followed by a desired outcome). Therefore, if the stimulus triggers the instrumental behavior in a later transfer test, this effect would have to be mediated by the representation of the desired outcome. That is, as the stimulus and the behavior never occurred together in the training phase, this effect cannot be regarded as a direct effect of stimulus-response (S-R) associations (Wood & Rünger, 2016) but has to be mediated by the goal representation.

Recently, this PIT paradigm has been applied to humans as well (Cartoni et al., 2016). Usually, specific PIT tests in humans require participants to perform two responses (e.g., pressing a left or right key) that produce two desirable outcomes or rewards (e.g., obtaining chocolates or crisps) to acquire response-outcome (R-O) associations in the instrumental learning phase. Furthermore, in the Pavlovian learning phase, participants learn unique stimulus-outcome (S-O) associations between two Pavlovian stimulus cues and the two outcomes. In the transfer test, it is tested whether participants’ responses are facilitated (e.g., more frequent, faster, or more accurate) when the stimulus cue and the response are associated with the same outcome, especially when the outcome that is shared by the cue and response is valuable to the person in the context at hand (Qin et al., 2021). Accordingly, the specific PIT effect can be used to demonstrate cue-based motivational control over goal-directed behavior in humans (Mahlberg et al., 2021).

In the present paper, we test an important prediction based on the notion that cues can motivate and control goal-directed behavior: If such motivational control is dependent on the value of the outcome, such control should be stronger for more valuable outcomes. While value has been successfully manipulated before (Qin et al., 2021, 2023) using the monetary reward of different value (e.g., 5 vs. 50 cents coins), here we focus on a universal property of outcomes: the fact that outcomes can satisfy multiple needs or higher order goals (i.e., multifinality; Kruglanski et al., 2002). For instance, although a specific action could be regarded as producing a single outcome (e.g., obtaining a snack to satisfy one’s appetite), actions can also be perceived as being instrumental in satisfying different needs or attaining multiple goals (e.g., obtaining a snack can satisfy appetite but can also serve as a present for a friend). Thus, by taking the hierarchical nature of goal-directed behavior into account (Carver & Scheier, 1981; Gallistel, 1985; Kruglanski et al., 2002; Vallacher & Wegner, 1987) and building on the notion that multi-functional objects are experienced as more valuable compared to single-functional objects (e.g., Brannon & Soltwisch, 2017; Ozcan & Sheinin, 2015), we examine whether the PIT effect is stronger when specific outcomes of actions (such as food) serve multiple outcomes.

People may find multi-functional objects more desirable than single-functional objects as multi-functionality (in comparison to single-functionality) renders behavior inherently more flexible and offers more degrees of freedom in responding to opportunities and demands posed by the social and physical environment (Bijleveld & Aarts, 2014; Kruglanski et al., 2015; Mikhalevich et al., 2017). Specifically, single-purpose objects put severe constraints on usability of the object. In contrast, multi-purpose objects allow for more choice, such as deciding when, how, and where to use the object (Zhang et al., 2022). According to the theory of self-determination, people have an innate need to act autonomously and therefore appreciate personal freedom of choice (i.e., the need for autonomy; Deci & Ryan, 1985; Ryan & Deci, 2000; Ryan & Deci, 2017). Having personal freedom of choice thus increases the desirability of goal-directed behavior and motivates people to engage in it. Single-functional objects, then, may be perceived to be less valuable than multi-functional objects because single-functionality forces the person to use the object in one way, while multi-functional objects offer more freedom.

In line with the notion of the relationship among personal freedom of choice, flexibility, and the value of objects, consumer psychology studies suggest that consumers prefer multi-functional products over single-functional products and consider multi-functional products to be more valuable (e.g., Brannon & Soltwisch, 2017; Ozcan & Sheinin, 2015). Similar effects have been found in the context of goal-means relations. Multi-final (vs. uni-final) means can attain more than one goal simultaneously. Such means or subgoals have an advantage over uni-final ones because they are considered to have greater overall value (Chun & Kruglanski, 2005; Orehek et al., 2012). The preference for multi-functional products is also reflected in consumers’ purchase intentions (Arruda Filho & Brito, 2017; Han et al., 2009). For example, Arruda Filho et al. (2017) showed participants different mobile phones that either included an environmental-friendly function or not (e.g., a solar energy recharge system). They found that participants’ purchase intention was stronger when the product had an additional function. Moreover, recent empirical research demonstrates that people value the freedom to choose and prefer choosing themselves over having a choice made for them (Shoval et al., 2022). Together, these studies suggest that multi-functional objects should be associated with higher perceived value than single-functional objects.

To summarize, existing studies on autonomy and consumer psychology have indicated that multi-functional outcomes should be perceived as having higher value. Given that outcome value plays an essential role in moderating the sensitivity of cue-based goal-pursuit (e.g., Qin et al., 2021), cues associated with multi-functional outcomes may benefit goal-directed behavior more in a cue-based goal-pursuit context. Hence, an important question that remains to be answered is whether cues associated with multi-functional outcomes are more effective in facilitating goal-directed behavior than cues linked with single-functional outcomes.

Testing this effect is crucial since it sheds light on how an individual’s representation of outcomes plays a role in the environmental control of goal-directed behavior. Human beings can pursue more abstract or high-level goals, and such processes can also be guided by environmental cues (also see: Qin et al., 2023). Here we investigate this higher level of abstraction by focusing on actions that are instrumental in obtaining outcomes that are desirable in multiple ways. This examination would offer a unique test of whether complex human goal-pursuit, especially abstract or high-level goals, can be studied testing for classical learning mechanisms (e.g., FeldmanHall & Dunsmoor, 2018). This exploration could serve as a significant point of reference for future studies on connecting fundamental learning processes (e.g., R-O and S-O associations) with the pursuit of high-level goals (Custers, 2023).

We report two experiments that examine whether cues can control goal-directed behavior. Specifically, we test whether cues referring to objects presented as multi- versus single-functional evoke stronger PIT effects, which represent stronger goal-directed behavior facilitated by cues (Mahlberg et al., 2021). Specifically, we relied on the cue-based forced-choice response time PIT paradigm (Qin et al., 2021, 2023) to test response facilitation upon exposure to outcome cues. First, participants were taught to press two different keys (left or right) to earn a snack they liked in the instrumental learning phase. We used one snack to manipulate the multi-functionality of the same snack without confounding the actual value or other features of different snacks. In Experiment 1, the snack was framed as serving only one single purpose on one condition. In the other condition, the snack was framed without such constraints. In Experiment 2, we further aimed to replicate Experiment 1 by explicitly addressing the role of multi-functionality in terms of perceived freedom of choice. In the Pavlovian learning phase, they learned to associate the single- or multi-functional snack with two different cues. In a final test phase, we exposed participants to the two Pavlovian cues just before executing one of the two responses. This setup allows us to test whether the PIT effect is stronger when the snack is not constrained and thus could serve multiple purposes compared to the single-functional snack cue.

## Experiment 1

The purpose of the first experiment is to provide initial support for the idea that specific PIT effects mainly show up for multi-functional objects. Multi-functionality was manipulated by stressing that one of the candy bars had to be consumed directly after the experiment in the lab (single-functional condition). The other candy bar could be taken home, thus implying that participants were allowed to do with it whatever they wanted (multi-functional condition). Based on the reasoning that multi-functionality increases the perceived value of objects, we examined whether participants’ responses were facilitated by Pavlovian cues associated with the multi-functional candy bar versus the cues associated with the single-functional candy bar.

## Method

### Participants and design

Aiming to detect a medium effect size (*η*_*p*_^*2*^ = 0.10, based on the previous study by Qin et al., 2021) with a power of 80%, we used 3 measurements for the 2 × 3 within-subjects design test and epsilon = 1 (Faul et al., 2007). The power analysis revealed that at least 46 participants were needed. We decided to recruit 5 more participants concerning the possible dropout. Finally, we recruited 51 undergraduate students (21 males; mean age 21.86 (*SD* = 1.80)) by posting advertisements targeting English-speaking students under the age of 40. Participants participated in the experiment where two different responses and two different cues could either be related to an object framed as single or multi-functional. This resulted in a 2 (Response outcome: single-functional object vs. multi-functional object) x 3 (Cue outcome: neutral vs. single-functional vs. multi-functional) repeated measures design. The neutral cue was used as a baseline to control for differences between the speed of single-functional object responses and multi-functional object responses. Participants received a fixed amount of €1 show-up payment. Moreover, they could earn two extra candy bars, one for consuming immediately after the experiment (single-functional outcome) and one for taking home to do anything they wanted with it (multi-functional outcome).

### Apparatus and materials

**Fig. 1:**
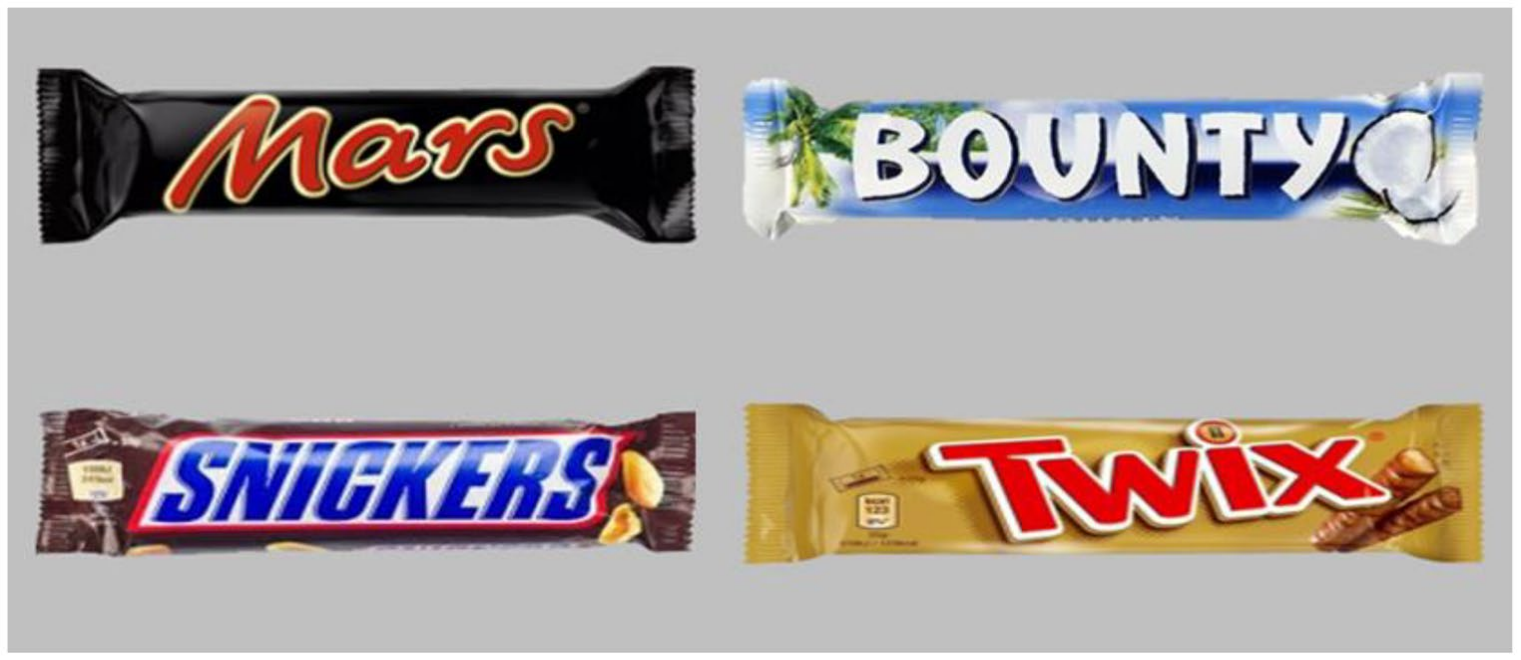
snacks used as the reward outcome

The experiment was conducted in a soundproof cubicle equipped with a computer monitor (1920*1080 pixels) and a standard keyboard. MATLAB’s Psychophysics Toolbox Version 3.0.10 was used to present the tasks (Brainard, 1997). At the beginning of the experiment, participants could select one snack from four candy bars (Fig. 1) as their reward. A grey square (RGB 192 192 192, visual angle 6.60˚), three figures (i.e., a ‘star’, a ‘moon’, and a ‘cloud’ visual angle 6.60˚) and two-colored frames (i.e., yellow, RGB 255 255 0 and blue, 0 0 255 visual angles 6.86˚) appeared in the experiment. The single and multi-functional snacks were represented by a full-color image of a selected snack (visual angles 6.60˚) with the words ‘NOW’ and ‘HOME’ printed, respectively. The word ‘NOW’ was used to refer to the single function (consume the snack), and the word ‘HOME’ was used to refer to the multi-functions (take it home and do whatever they like with it).

### Procedure

Upon arrival at the laboratory, participants signed the informed consent, and the experimenter told participants that this experiment aims to detect how fast people can react to visual stimuli. Before the experiment started, participants had to indicate which out of four types of candy bars they would like to earn (see Fig. 1) as rewards. Specifically, they were informed that they had to collect (a non-specified number of) points to earn this reward by performing two experimental (instrumental and Pavlovian learning) tasks. This apparent progression in earning points was assumed to increase the motivation to perform well (Pierce et al., 2003; Locke & Braver, 2008).

They also learned that they could earn two of their candy bars as snacks in total, but one could be consumed immediately, and the other could be taken home so they could do whatever they like with it. We refer to this condition as the single- and multi-functional outcome, respectively.

Next, they filled out a questionnaire to check whether participants valued the multi-functional snack more than the single-functional snack. Participants responded to six items (3 items for each type of snack) to assess their liking, willingness to spend effort, and motivation to obtain the snacks. The self-report items were measured on a 5-points Likert scale (see Supplemental Materials for details). After the questionnaire, the experiment started.

The experimenter stayed in the cubicle during the entire experiment to note their performance. The experiment contains four phases: a demonstration phase, an instrumental learning phase, a Pavlovian learning phase, and a test phase.

***Demonstration phase.*** During this phase, participants performed the speeded response task that was also administered during the test phase to familiarize them with the procedure of the task. Participants performed 42 randomly presented trials in total.

***Instrumental learning phase.*** Participants learned that they earned points for obtaining the single or multi-functional snack by producing two different motor responses. Participants first practiced 20 trials (block 1), followed by 20 real trials (block 2). The trials in the practice and the actual task were randomly presented, and each condition (i.e., the single-functional snack response and the multi-functional snack response) was repeated 10 times in each block. The trial procedure is depicted in Fig. 1 (panel A): Each trial started with a grey square for 1–3 seconds (random time interval), then a yellow or blue frame indicated to press the left or right key. Participants could earn points for getting the single-functional snack by correctly pressing the (left) ‘s’ key (yellow frame) and the multi-functional snack by correctly pressing the (right) ‘k’ key (blue frame); colored frames were counterbalanced across participants. After a correct keypress, the single-functional or multi-functional outcome was presented for 1 second (i.e., a picture of the single-functional snack titled ‘NOW’ or the multi-functional snack titled ‘HOME’), meaning participants earned points for the single-functional or multi-functional snack. If participants made a wrong keypress, they saw a red cross. The snack picture displayed the word ‘NOW or ‘HOME to support participants in keeping the single vs. multi-functional outcome in mind. To encourage participants to process the outcome information carefully, they had to speak out ‘snack for now’ or ‘snack for home’ upon seeing the snack (Qin et al., 2021, 2023, for a similar procedure). The experimenter noted whether participants spoke out the correct outcome at the moment.

Participants did not know in advance how many points they could earn. They also did not know how many trials they had executed and how many trials they had to do. After the task, all participants were informed that they performed well. We decided to inform all participants that they earned 200 points (suggesting they made progress in obtaining the snacks). Actual earnings thus were independent of the keypress performance.

***Pavlovian learning phase***. In this phase, participants learned that they could earn points for the single and multi-functional snacks in a cue–outcome learning task. Participants performed 40 trials (2 blocks); the first half was practice trials (block 1), and the second half was the actual trials (block 2). The practice and the actual trials were randomly presented, and each condition (i.e., the single-functional snack cue and the multi-functional snack cue) was repeated 10 times in each block.

**Fig. 2:**
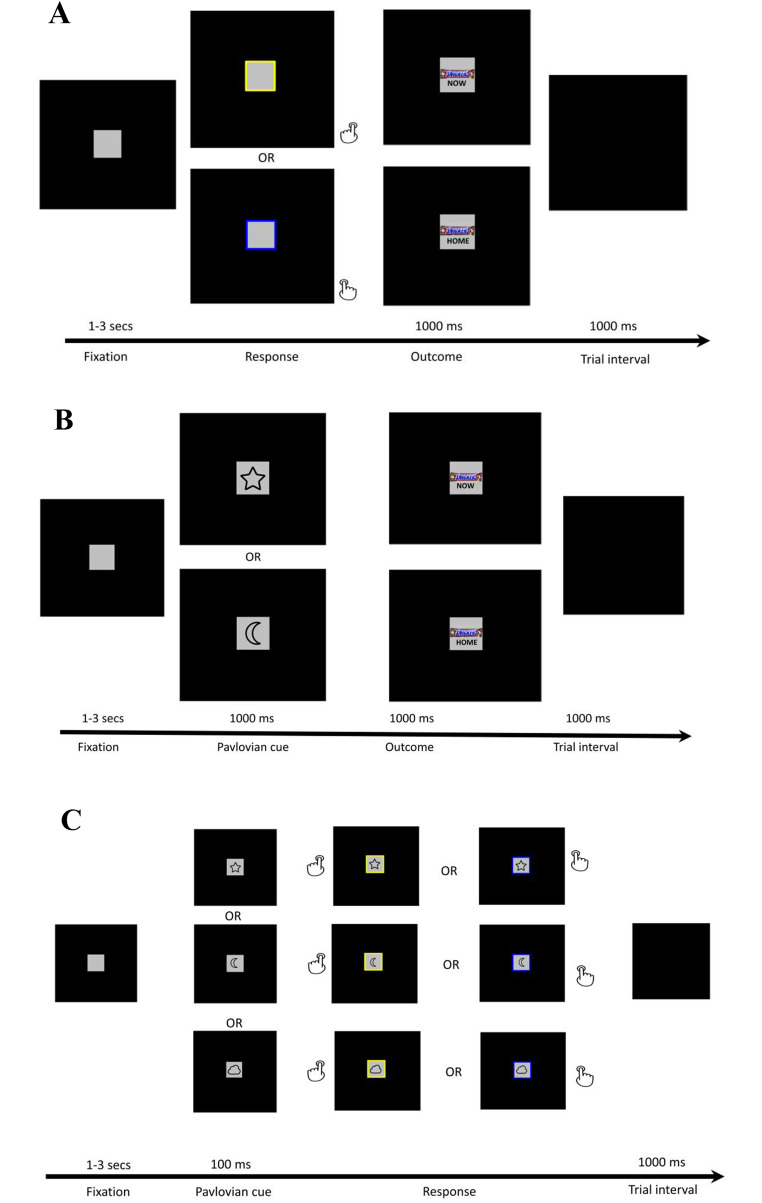
Flowchart of the correct response in Instrumental learning phase (A), Pavlovian learning phase (B), Test phase (C)

The trial procedure was as follows (see Fig. 2, panel B): A grey square appeared for 1–3 seconds (random time interval), then one of two cues (e.g., a ‘star’) appeared for 1 second. Participants earned points for the single-functional snack by speaking out ‘snack for now’ when they saw a ‘star’ and points for the multi-functional snack by speaking out ‘snacks for home’ when they saw a ‘moon’ (the particular S-O mapping was counterbalanced across participants). The experimenter took notes on whether they spoke out the correct outcome in response to the cues. The picture of the single-functional and multi-functional snack (NOW snack or HOME snack) was presented when they spoke out the corresponding outcome. Participants only earned points for the actual task.

Like in the instrumental learning phase, participants did not know how many points they could earn, how many trials they had executed, and how many trials they had to do in the actual task. After the task, they were told how many points they had earned. We again decided to give all participants the number of points. Hence, they were informed that they performed well and earned 200 points. Actual earnings points thus were independent of performance. Accordingly, all participants learned that they had enough points to receive both the single and multi-functional snacks in both tasks.

***Test phase.*** Participants were informed that they could not further earn points in this phase. They were asked to respond as quickly and accurately as possible with the left or right keypress in a series of trials. The trial procedure of the speeded response task was taken from Qin et al. (2021) and looked as follows (see Fig. 2, panel C): Each trial started with a grey square, followed by one of the three cues (‘star’ or ‘moon’ or ‘cloud’) which appearing inside the grey square after a 1–3 seconds (randomized time interval). After 100ms, a colored frame appeared on the computer screen surrounding the grey square, thus prompting participants to press the left or right key (counterbalanced). The Pavlovian cue remained on the screen until a response was given.

In the test phase, the cues (‘star’ and ‘moon’) that were learned to be associated with single-functional versus multi-functional snacks (or vice versa) were combined with the responses (pressing ‘s’ and ‘k’ keys) that were also learned to be associated with single-functional versus multi-functional snacks. To iterate, then, a value-based specific PIT effect emerges when the multi-functional snack cue speeds up the multi-functional snack response, while such a speed-up effect is not expected for single-functional snack responses that are preceded by single-functional snack cues. A third neutral cue (e.g., a ‘cloud’) served as a baseline condition. This cue was not learned to be associated with any of the outcomes, thus allowing us to check for response time differences between single-functional and multi-functional snack responses that are independent of PIT effects. There were 120 trials (4 blocks) in total. The trials were randomly presented, and each condition was repeated 5 times in each block.

After the test phase, all participants received the two candy bars, one they had to consume immediately and one they could take home. Participants consumed the former when they received it and took the latter with them.

### Data preparation and analyses

We trimmed the RT data of correct responses in the test phase for outliers as in previous studies (Qin et al., 2021, 2023). Specifically, RTs from incorrect responses and RTs that were slower or faster than 3 SD of the participants’ mean were removed from analyses (4.6% of the RT data). Since the RT and accuracy data were not normally distributed, we performed a reciprocal transformation (i.e., 1/x) to normalize the distributions (for details, see Supplemental Materials). We used the transformed data for further tests^1^.

We analyzed the RTs data as in previous studies (Qin et al., 2021, 2023). We performed a planned contrast to the RT difference in three cue conditions using an F-test with partial eta squared (*η*^*2*^_*p*_) as effect size, which is reported with a 90% CI (Furr & Rosenthal, 2003; Rosenthal & Rosnow, 1985). Participants should respond more readily when the cue and the response predict the same desirable outcome in specific PIT effects. Accordingly, if representing the snack from a multi-functional (vs. single-functional) point of view enhanced the subjective value of the snack, then the PIT effect should mainly occur in the multi-functional outcome condition. This means that the RT difference between the multi-functional and single-functional outcome response should be larger in the multi-functional cue condition compared to the RT difference in the neutral cue and the single-functional outcome cue condition. Since the single-functional representation is expected not to enhance the value of the outcome when compared to the multi-functional representation, the responses to the neutral and single-functional cues will not differ.

To test this, we subjected the RT differences (single-functional outcome responses minus multi-functional outcome responses) to a repeated ANOVA with neutral, single-functional, and multi-functional cues as a within-subject factor. Note that a negative RT difference represents a facilitation effect for responses that lead to single-functional outcomes, and a positive one represents a facilitation effect for responses that lead to multi-functional outcomes. We tested effects according to the following contrast: -1 for the RT difference in the neutral cue condition, -1 for the RT difference in the single-outcome cue condition, and + 2 for the RT difference in the multi-functional outcome cue condition. Compared to the neutral cue, then, the multi-functional outcome cue should speed up the multi-functional (vs. single-functional) outcome response, while the single-functional outcome cue does not (or to a lesser extent) speed up the single-functional (vs. multi-functional) outcome response. The same approach was also applied to the accuracy data, but the contrast coding weight was reversed because participants should respond more accurately when the cue shares the identical outcome representation with the response. Note that a positive accuracy difference score represents more accurate responses toward single-functional outcomes, and a negative one indicates more accurate responses that lead to multi-functional outcomes.

To analyze the questionnaire data, we conducted three t-tests (2-tailed) to compare the self-report scores of liking, willingness to spend effort, and motivation to obtain the single-functional and multi-functional snacks.

## Results

### Reaction times

**Fig. 3:**
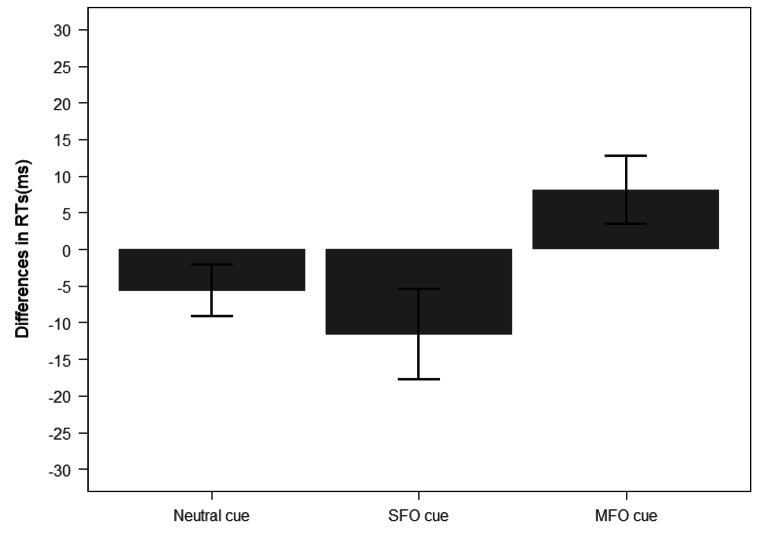
Experiment 1 RT difference in the three cue conditions of the test phase (Error bar represents one standard error). SFO represents the single-functional outcome, and MFO represents the multi-functional outcome. Note: A positive score represents faster multi-functional responses, and a negative score represents faster single-functional responses

The pattern of reaction time differences in each cue condition is presented in Fig. 3. The planned contrast was significant (*F* (1, 50) = 5.94, *p* = .018, *ƞ*_*p*_^*2*^ = 0.11 [0.010; 0.253]). In line with predictions, the RT difference score between the multi-functional and single-functional outcome responses is positive in the multi-functional cue condition compared to the other two conditions, indicating that multi-functional cues facilitated multi-functional outcome responses. Furthermore, whereas the RT difference score between the multi-functional and single-functional outcome responses is negative in the single-functional cue condition (suggesting that single-functional cues facilitated single-functional outcome responses), the RT difference does not seem to differ between the neutral and single-functional cue conditions.

### Accuracy

**Fig. 4:**
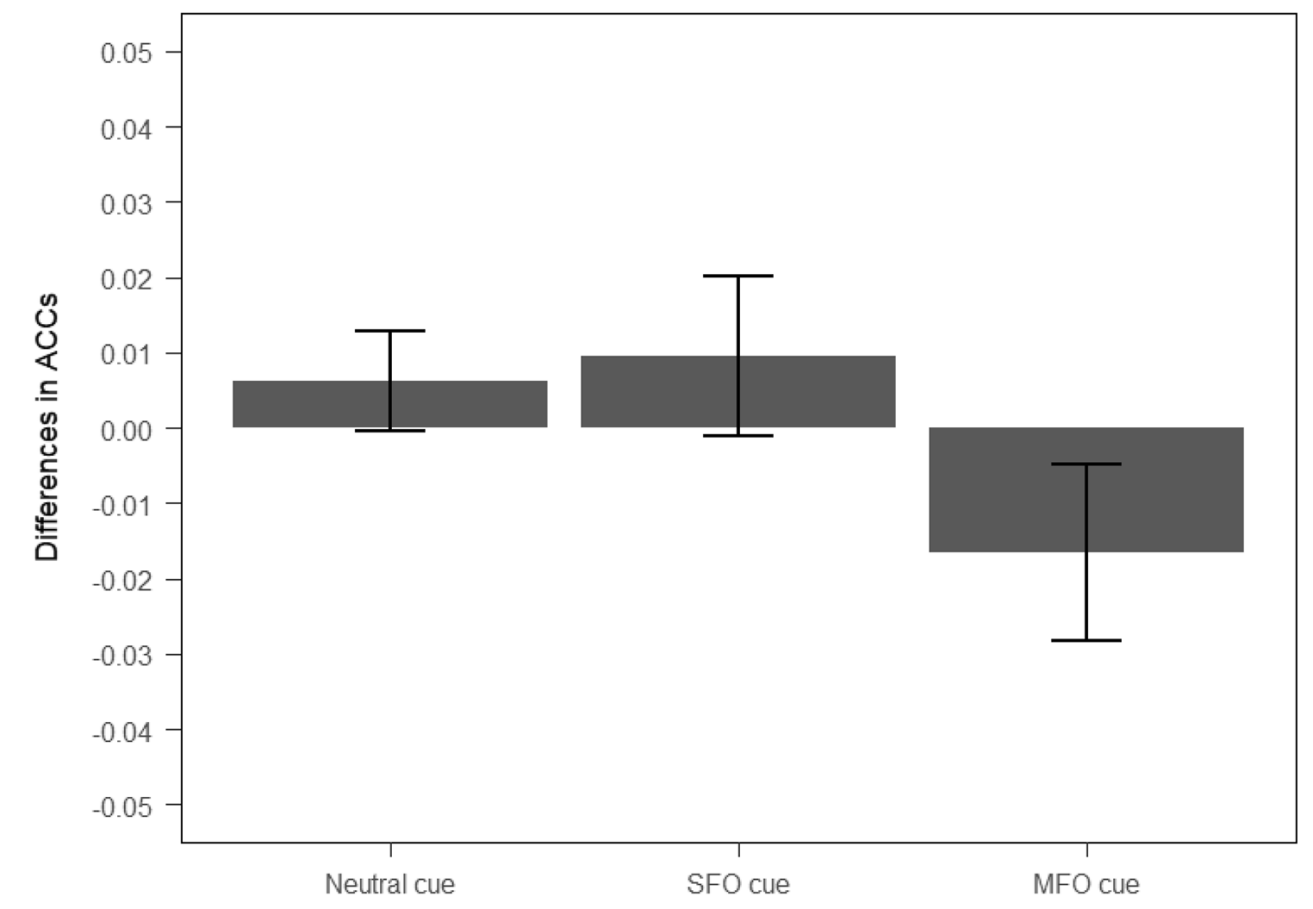
Experiment 1 accuracy difference in the three cue conditions of the test phase (Error bar represents one standard error). SFO represents the single-functional outcome, and MFO represents the multi-functional outcome. Note: A negative score represents more accurate multi-functional responses, and a positive score represents more accurate single-functional responses

Figure 4 shows the accuracy difference pattern in the three cue conditions. The planned contrast yielded no significant effect (*F* (1, 50) = 2.65, *p* = .110). Although not significant, please note that the accuracy pattern shows that participants responded more accurately to the multi-functional outcome response (vs. single-functional outcome response) when encountering the multi-functional outcome cue. This suggests that the RTs effect cannot be easily explained by a speed-accuracy trade-off.

### Self-report data

The separate t-tests indicated that participants liked the multi-functional snack more (*M* = 3.94, *SD* = 1.08) than the single-functional snack (*M* = 3.29, *SD* = 1.03, *t* (50) = 2.92, *p* = .005, *Cohen’s d*_*z*_ = 0.41). Furthermore, they were willing to spend more effort to get the multi-functional snack (*M* = 3.37, *SD* = 1.26) compared to the single-functional snack (*M* = 2.75, *SD* = 1.16, *t* (50) = 3.11, *p* = .003, *Cohen’s d*_*z*_ = 0.44). They also reported higher motivation to get the multi-functional snack (*M* = 3.59, *SD* = 1.20) compared to the single-functional snack (*M* = 3.18, *SD* = 1.14, *t* (50) = 2.10, *p* = .041, *Cohen’s d*_*z*_ = 0.29). In short, the self-reports clearly show that the multi-functional (vs. the single-functional) candy bar was perceived as more valuable.

## Discussion

The results of Experiment 1 provide initial evidence that cue-based goal-directed behavior is more likely to materialize for behaviors that are represented as having multi-functional (vs. single-functional) outcomes. Taking the RTs of single-functional and multi-functional snack responses to neutral cues as a baseline, the significant planned contrast of RTs indicates that cues associated with the multi-functional snack facilitated multi-functional snack responses, while cues associated with the single-functional snack did not facilitate single-functional snack responses.

It is important to note that the manipulation regarding the multi-functionality remained rather implicit. The single function of eating the snack immediately after the study explicitly forced participants to use the object in one way. However, we do not know whether participants experienced freedom of choice and considered other purposes than eating when taking the snack home. In other words, whereas participants represented the ‘NOW’ snack in terms of being forced to use it in one way, it can be questioned whether they represented the ‘HOME’ snack as an object they could use in different ways and thus were free in using it. If the two snacks do not differ in multi-functionality representations, our findings could be ascribed to the higher likeability of taking the snack home and not to multi-functional value per se. To examine the multi-functionality aspect more thoroughly, we conducted a second experiment where the multi-functionality of the snack manipulation was designed to be very explicit in terms of being forced or free in using the same snack in one way or several ways, respectively.

## Experiment 2

To corroborate the findings of Experiment 1, we more strongly relied on the need for autonomy (Ryan & Deci, 2000, 2006), which explicitly deals with restricted freedom of choice or not and is at the essence of human motivation. Participants could again earn a candy bar snack, but we explicitly enforced the single-functional snack by telling participants that they could only do one thing with it, namely eating it after the experiment. Furthermore, for the multi-functional snack, we made it explicitly clear that the candy bar could be used for several purposes after the experiment by providing three example options: eating it themselves, giving it away to another person, or giving it back to the experimenter to receive money for it in return. We did not make any references about taking the snack home. Accordingly, we made clear that participants were *forced* to use one snack only in one way (single-functional object condition), while they were *free* to use the other snack in multiple ways (multi-functional object condition). Building on the findings of Experiment 1, and research on personal autonomy and freedom of choice, we tested whether participants’ responses were facilitated by Pavlovian cues associated with the multi-functional candy bar versus the cues associated with the single-functional candy bar. This experiment was pre-registered in OSF^2^.

## Method

### Participants and design

We increased the sample size to obtain a more sensitive measure for detecting a specific PIT effect, and we recruited 60 participants (14 males, mean age 25.85 SD = 6.78). Data from two participants were excluded since one had excessively low accuracy in the test phase (< 3 SD from the sample mean), and the other participant responded extremely slow (> 3 SD from the sample mean). The remaining 58 participants were subjected to the 2 (Response outcome: single-functional vs. multi-functional) x 3 (Cue outcome: neutral vs. single-functional vs. multi-functional) repeated measures design experiment. They received a fixed amount of 10 Euros^3^ as a participation fee before the experiment. Like Experiment 1, they could earn two candy bars; one they were forced to consume (single-functional), and one were free in whatever they wanted to do with it (multi-functional).

### Apparatus and materials

Apparatus and materials were the same as in Experiment 1 except for the image of outcomes that appeared in the learning phases and the questionnaire. In direct correspondence with the concept of personal freedom of choice, we replaced the text below the candy bar image with ‘FORCED’ and ‘FREE’ to represent the single and multi-functional outcomes, respectively. Accordingly, we revised the questionnaire to capture the forced and free wording, including 7 items. We measured liking, attractiveness, and desire to take each snack home. As a seventh item, we asked participants to indicate which of the two snacks they preferred. The self-report items were measured on a 9-points Likert scale since it might produce a larger comparative variance to reveal differences between items, and it might increase reliability compared to the 5-points Likert scale (Finn, 1972; Oaster, 1989) (see Supplemental Materials).

### Procedure

The procedure was mostly the same as Experiment 1, but this experiment was run during the Covid-19 pandemic, and specific precautions were taken. We followed the Covid-19 protocol of Utrecht University when running the study. Specifically, the experimenter kept a distance of 1.5 m from participants during the entire experiment. Furthermore, the experimenter did not stay with participants in the same cubicle but used video and microphones to communicate with participants and monitor the progress of the experiment. At the end of the experiment, participants filled out the questionnaire. Finally, they received the two snacks. One of the snacks they had to consume, and for the other, they were reminded of the multiple options, including the option to exchange the snack for a monetary reward. In total, 34 participants exchanged the snack for money (i.e., €0,50), and 26 participants chose to do something else with it.

### Data preparation and analyses

Like Experiment 1, we trimmed the RT data of the correct responses in the test phase for outliers (3.9% of RT data), which is defined as slower or faster than 3 SD of each participant’s mean (Lachaud & Renaud, 2011). Since the RTs and the accuracies were non-normally distributed, we performed a reciprocal transformation for both the RTs and the accuracies. Following up on the findings of Experiment 1, we predicted that specific PIT effects should only be observed in the multi-functional outcome cue condition. We, therefore, calculated the difference for the RT and the accuracy data and analyzed them with the same approach as Experiment 1.

For analyzing the self-report data, we conducted three paired t-tests (2-tailed) to compare the self-report scores of liking, attractiveness, and to what extent participants wanted to take the single-functional and the multi-functional snack home, respectively. We also did a one-sample t-test on the preference item to test which snack participants preferred.

## Results

### Reaction times

**Fig. 5:**
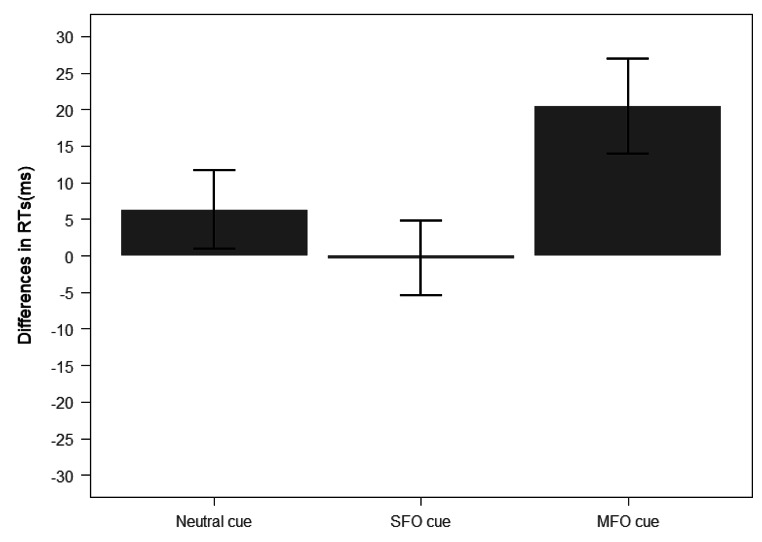
Experiment 2 RT difference in the three conditions of the test phase (Error bar represents one standard error). SFO represents the single-functional outcome, and MFO represents the multi-functional outcome. Note: A positive score represents faster multi-functional responses, and a negative score represents faster single-functional responses

Figure 5 shows the pattern of RT difference in the three cue conditions. The planned contrast yielded a significant contrast effect (*F* (1, 57) = 6.12, *p* = .016, *ƞ*_*p*_^*2*^ = 0.10 [0.010; 0.232]). The pattern of means indicated that in the multi-functional outcome cue condition, the RT difference score between the single-functional outcome response and the multi-functional outcome response is larger and positive compared to the RT difference score in the other two conditions. The RT difference did not seem to differ between the neutral and single-functional cue conditions.

### Accuracy

**Fig. 6:**
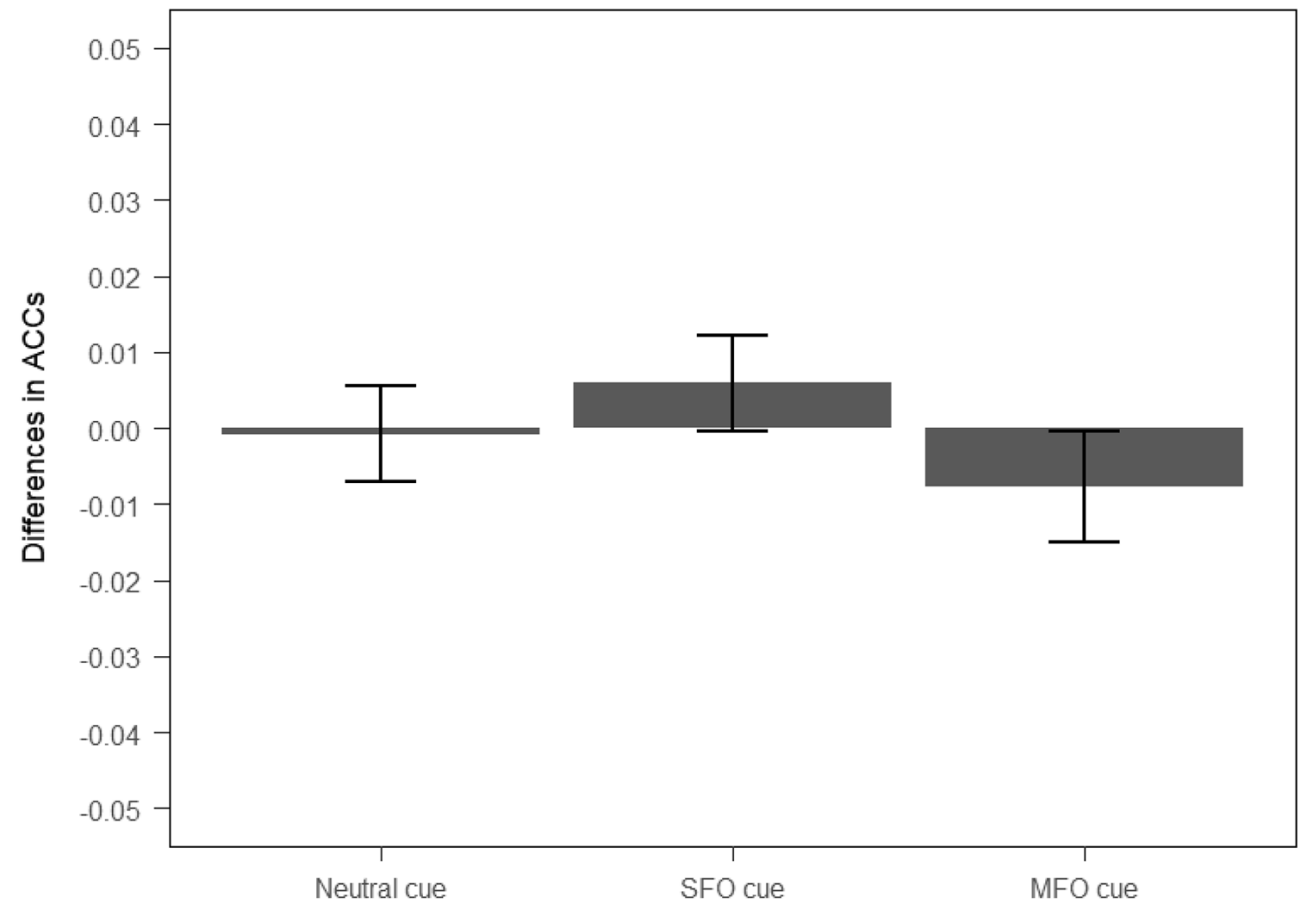
Experiment 2 accuracy difference in the three conditions of the test phase (Error bar represents one standard error). SFO represents the single-functional outcome, and MFO represents the multi-functional outcome. Note: A negative score represents more accurate multi-functional responses, and a positive score represents more accurate single-functional responses

The planned contrast for accuracy difference in the three cue conditions was not significant (*F* (1, 57) = 1.49, *p* = .227). The accuracy difference pattern is presented in Fig. 6.

### Self-report data

The results indicated that participants liked the multi-functional snack (*M* = 6.62, *SD* = 2.12) more than the single-functional snack (*M* = 5.12, *SD* = 2.33, *t* (57) = 4.54, *p* < .001, *Cohen’s d*_*z*_ = 0.60). They also felt the multi-functional snack (*M* = 6.84, *SD* = 1.72) was more attractive compared to the single-functional snack (*M* = 4.62, *SD* = 2.38, *t* (57) = 7.15, *p* < .001, *Cohen’s d*_*z*_ = 0.94). Importantly, we did not find a difference in how much participants liked to take the multi-functional snack (*M* = 5.98, *SD* = 2.98) or the single-functional snack home (*M* = 5.76, *SD* = 2.84, *t* (57) = 0.44, *p* = .661), suggesting that we ruled out the possibility that the multi-functional object is merely represented as a snack that one likes to take home. Additionally, the one-sample t-test, which examined whether participants favored the multi-functional snack compared to the single-functional snack by comparing the score with the median value of 5, indicated that participants had a strong preference for the multi-functional snack (*M* = 8.17, *SD* = 1.44, *t* (57) = 16.77, *p* < .001, *Cohen’s d*_*z*_ = 2.20). Taken together, these results offer clear evidence that participants valued the multi-functional snack more than the single-functional one.

## Discussion

The findings of Experiment 2 replicated the value-based specific PIT effect observed in Experiment 1; cues associated with the multi-functional snack increased the expected difference in response times between multi-functional and single-functional snack responses, while the neutral cue and the cue associated with the single-functional snack did not produce these differences in response times. In contrast to Experiment 1, the multi-functionality manipulation was not confounded with where and when to use the snack. In Experiment 2, we made it more explicit that a snack served only one purpose or multiple purposes by listing examples of such purposes. It was, therefore, clear to participants that they were *forced* to use one snack in one way and were *free* to use the other snack in different ways.

## General discussion

The present study examined whether cues can gain motivational control over goal-directed behavior by exploiting the PIT paradigm in a forced-choice reaction time test. According to specific PIT, cues can trigger outcome-related actions when such outcome is of personal value, even though a person has not directly learned to perform the action in response to the cue. So far, PIT research has focused on actions with one single functional outcome. Research on the hierarchical organization of human behavior suggests that actions can serve multiple outcomes and goals at different levels of decision-making, offering flexibility and degrees of freedom in engaging in goal-directed behavior (Carver & Scheier, 1981; Kruglanski et al., 2002; Vallacher & Wegner, 1987). Hypothesizing that actions that can serve multiple outcomes are perceived to be more valuable, we tested whether PIT effects are stronger for actions serving multiple outcomes. Overall, our findings indicate that specific PIT effects are more pronounced for actions related to objects that serve multiple purposes than for objects that serve only one purpose, suggesting that a multi-functionality context changes PIT effects by increasing the motivational strength by which Pavlovian cues can trigger goal-directed behavior.

It is important to note that previous research established the motivational nature of specific PIT for goal-directed actions in a setting where two actions each had one single (low or high-value) outcome. Furthermore, these outcomes consisted of objects (e.g., cucumber or chocolate) that differ in perceptual information (e.g., Alarcón et al., 2018; Alarcón & Bonardi, 2016; Qin et al., 2021; Watson et al., 2016). Whereas the observed PIT effects in earlier research may result from the differences in motivational relevance attached to the objects, other features of the stimulus objects (e.g., ease of processing, familiarity) might also contribute to the effects. In the present study, we used one single stimulus object (e.g., a candy bar) and manipulated the psychological meaning of the object. We framed the very same object as having one function or multiple functions. As earlier research indicates, multi-functional objects offer more freedom in acting and achieving different goals and are therefore perceived as more valuable (Bijleveld & Aarts, 2014; Han et al., 2009; Kruglanski et al., 2015; Ryan & Deci, 2006). This notion was corroborated by the checks in the present studies. In line with an outcome value-based account, stressing the multi-functionality of an object rendered the same object more prone to PIT.

Our findings suggest that the PIT forced-choice task can separate cue-based goal-directed behavior with multiple outcomes versus one single outcome. Although encouraging, a few important notes are in place to put these effects in broader perspectives. First, in the present study, the snack was selected based on participants’ personal preferences; hence, the snack should be associated with experienced pleasure. Earlier research has found specific PIT effects for pleasurable objects (e.g., Allman et al., 2010). Considering this, a rather notable finding in the present study is that a specific PIT effect did not clearly show up in the single outcome condition representing a pleasurable object. Two possible reasons may account for the observed pattern. Firstly, although not investigated, it is possible that in previous research, participants considered the objects (e.g., food and drinks) as having multiple functions. Research suggests that people differ in how they represent their actions in terms of different goals (van der Weiden et al., 2010; Vallacher & Wegner, 1987). Hence, earlier studies might have established PIT effects partly due to the perceived multi-functionality of the objects obtained by the instrumental actions.

A second possibility pertains to the manipulation and experimental design of the present study. We forced participants to consider one initially pleasurable object with only one functionality, which decreased their experiences of personal autonomy and freedom of choice. Because freedom of choice is essential in determining individuals’ internal motivation (Deci & Ryan, 1985; Ryan & Deci, 2000), it is possible that the single-functional outcome completely lost its value because of the pain of losing freedom. Furthermore, using this manipulation in a within-subject design might have created a comparison between the two snacks and, as revealed by the self-reported checks, caused participants to consider the single-functional snack relatively less valuable than the multi-functional snack. Such considerations, then, might have overridden the initial pleasure experiences of the snack. Whereas comparisons between two objects are less likely to occur in a between-subject design, future research could explore whether the experimental design of testing can explain the absence of the specific PIT effect for single functional objects.

Furthermore, we wish to note that our study followed an outcome value comparison approach in which action, cues, and outcomes of different values (single vs. multi-functional) become associated because of two separate learning processes: Instrumental and Pavlovian learning. Whereas our outcome value comparison approach was able to demonstrate a value-based specific PIT effect, it might be informative to combine this approach with the devaluation approach (Dickinson & Balleine, 1994). According to this approach, stimuli that influence responses through the activation of goal representations should have less of an effect on behavior if the goal is rendered less valuable (i.e., devaluated). Specifically, one could create conditions that render outcomes less relevant or useful, which should mainly affect PIT effects for high-value outcomes. For instance, informing participants that both snacks are expired should remove the PIT effect of the earlier represented multi-functional snack. Moreover, the outcome devaluation procedure can also be used in a reversed way, examining whether PIT effects show up when the value of the outcome is increased (Eder & Dignath, 2016). For example, one could inform participants that the single-functional snack can also be used in several ways, causing a PIT effect in the earlier represented single-functional snack condition. In general, integrating the outcome value comparison and devaluation approach allows for a full test in showing the dynamics of how cues trigger goal-directed behavior when values of goals come and go in the situation at hand (Aarts, 2007; Marien et al., 2013).

Finally, the current findings may have important implications for research on habits. Habits are often regarded as involuntary actions resulting from stimulus-response (S-R) links that operate automatically and rule out freedom of choice (Wood & Rünger, 2016). However, research using the PIT paradigm suggests that these effects can also be mediated by the activation of goal representations. As our research suggests that responses that serve multiple outcomes can have a stronger effect on behavior than responses that serve a single outcome, researchers may be prone to overestimate the habitual nature of multi-functional responses (cf., De Houwer, et al., 2018). To properly determine the habitual nature of behavior, especially in more applied and societal contexts, it may not only be important to consider whether there are goals that could mediate these effects, but also how many potential goals the behavior could serve (see Marien et al., 2019 for a more elaborate discussion).

To conclude, the present study shows that representing the same action-outcome in terms of a single-functional vs. multi-functional object alters how outcome-related actions respond to cues. In everyday life, people might experience freedom of choice when they represent their actions in terms of serving different goals according to the context in which they are relevant. For example, taking a soda from the fridge upon entering the kitchen can be represented as a means to satisfy thirst after sports but represented as an act of hospitality when friends come over to watch a movie. Previous research has examined the cognitive and motivational aspects of the process underlying the representation and control of goal-directed behavior (Aarts & Elliot, 2012; Ajzen & Kruglanski, 2019). However, less attention has been given to empirically addressing how goal-directed behaviors with multiple functions are causally linked to and triggered by the environment (but see Custers & Aarts, 2010). We hope that the present research may connect the study of multi-functionality, freedom of choice, and PIT to understand better how actions that can serve different goals can become under the control of the environment.

## Electronic supplementary material

Below is the link to the electronic supplementary material.

Supplementary Material 1

## Data Availability

The datasets generated during and/or analyzed during the current study are available in OSF repository, https://osf.io/vpfbt/?view_only=2293ed6bc1d7498d8d8159f34a5d763e.

## Footnotes

1 Although we used transformed data to feed the analysis, for clarifying the predicted pattern, figures of the RTs and the accuracies in both experiments were presented with untransformed data.

2 https://osf.io/e4rcj/?view_only=319a7cd9bd0a4bf7900105d7c6c75d87.

3 This amount was higher than experiment 1 because the Covid-19 measures made the experiment for participants more invasive with all the extra precautions that needed to be taken.
