# Supplementary material for: How the environment evokes actions that lead to different goals: the role of object multi-functionality in pavlovian-to-instrumental transfer
Source: Curr Psychol. 2023 Apr 11:1–14. Online ahead of print. doi: 10.1007/s12144-023-04612-2 (PMC10088748; doi:10.1007/s12144-023-04612-2)
Supplement: Supplementary file 1 — Supplementary Material 1 [file 12144_2023_4612_MOESM1_ESM.docx]

**Supplementary materials**

**The questionnaire used in Exp 1**

Please answer the following questions about the snack of choice.

Tick the box that fits best with your opinion.

1. To what extent would you like to eat the snack right now?

Not at all – not much – neutral – somewhat – very much

2. How much effort would you make to be able to eat the snack right now?

Not at all – not much – neutral – somewhat – very much

3. To what extent are you motivated to eat the snack right now?

Not at all – not much – neutral – somewhat – very much

4. To what extent would you like to take the snack home with you?

Not at all – not much – neutral – somewhat – very much

5. How much effort would you make to be able to take the snack home with you?

Not at all – not much – neutral – somewhat – very much

6. To what extent are you motivated to take the snack home with you?

Not at all – not much – neutral – somewhat – very much

## Exp 1 instrumental training RTs

### Descriptive analysis

Table 1 Descriptive analysis of Exp 1instrumental training

| Response | RT_mean | RT_sd | ACC_mean | ACC_sd |
| --- | --- | --- | --- | --- |
| SFO response | 464.633 | 92.634 | 0.984 | 0.042 |
| MFO response | 469.203 | 85.563 | 0.996 | 0.020 |

Note: SFO: Single-functional outcome; MFO: Multi-functional outcome

### Normality test

Table 2 Normality test of the instrumental training RTs

| items | w_value | p_value |
| --- | --- | --- |
| SFO response | 0.941 | 0.013 |
| MFO response | 0.933 | 0.007 |

Table 3 Normality test of instrumental training accuracy

| items | w_value | p_value |
| --- | --- | --- |
| SFO response | 0.421 | <0.001 |
| MFO response | 0.196 | <0.001 |

### RTs and ACC test

The results indicate that no difference was found on RTs^[[1]](#footnote-1)^ (*t* (50) = 1.16, *p* = .254) and accuracy (*t* (50) = 1.76, *p* = .084) in instrumental training phase.

## Exp 1 test phase RTs and ACC

### Descriptive analysis

Table 4 descriptive analysis of Exp 1 test phase

| Response | Cue | RT_mean | RT_sd | ACC_mean | ACC_sd |
| --- | --- | --- | --- | --- | --- |
| SFO response | Neutral cue | 403.191 | 50.999 | 0.979 | 0.046 |
| MFO response | SFO cue | 393.651 | 49.297 | 0.976 | 0.036 |
| SFO response | MFO cue | 405.237 | 49.558 | 0.958 | 0.065 |
| MFO response | Neutral cue | 408.819 | 53.253 | 0.973 | 0.037 |
| SFO response | SFO cue | 405.282 | 56.138 | 0.966 | 0.054 |
| MFO response | MFO cue | 397.128 | 50.955 | 0.974 | 0.047 |

### Normality test

Table 5 Normality test of the test phase RTs

| items | w_value | p_value |
| --- | --- | --- |
| SFO response & Neutral cue | 0.884 | <0.001 |
| SFO response & SFO cue | 0.941 | 0.013 |
| SFO response & MFO cue | 0.936 | 0.008 |
| MFO response & Neutral cue | 0.921 | 0.002 |
| MFO response & SFO cue | 0.937 | 0.009 |
| MFO response & MFO cue | 0.940 | 0.011 |

Table 6 Normality test of test phase accuracy

| items | w_value | p_value |
| --- | --- | --- |
| SFO response & Neutral cue | 0.531 | <0.001 |
| SFO response & SFO cue | 0.690 | <0.001 |
| SFO response & MFO cue | 0.677 | <0.001 |
| MFO response & Neutral cue | 0.707 | <0.001 |
| MFO response & SFO cue | 0.678 | <0.001 |
| MFO response & MFO cue | 0.601 | <0.001 |

**The questionnaire used in Exp 2**

1.How much do you like the ‘FORCED’ snack that you have to eat immediately?

1(Not at all)-2-3-4-5-6-7-8-9(Very much)

2.How attractive is the ‘FORCED’ snack to you?

1(Not at all)-2-3-4-5-6-7-8-9(Very much)

3.How much do you like the ‘FREE’ snack you can do with whatever you want?

1(Not at all)-2-3-4-5-6-7-8-9(Very much)

4.How attractive is the ‘FREE’ snack to you?

1(Not at all)-2-3-4-5-6-7-8-9(Very much)

5.Which one would you prefer? Please select a number that fits your answer?

1(‘Forced’ snack)-2-3-4-5-6-7-8-9(‘Free’ snack)

6.Would you like to take the ‘FORCED’ snack home?

1(Not at all)-2-3-4-5-6-7-8-9(Very much)

7.Would you like to take the ‘FREE’ snack home?

1(Not at all)-2-3-4-5-6-7-8-9(Very much)

## Exp 2 instrumental training RTs

### Descriptive analysis

Table 7 Descriptive analysis of Exp 2 instrumental training phase

| Response | RT_mean | RT_sd | ACC_mean | ACC_sd |
| --- | --- | --- | --- | --- |
| SFO response | 499.559 | 124.833 | 0.984 | 0.042 |
| MFO response | 492.462 | 110.812 | 0.988 | 0.033 |

### Normality test

Table 8 Normality test of the instrumental training RTs

| items | w_value | p_value |
| --- | --- | --- |
| SFO response | 0.853 | <0.001 |
| MFO response | 0.899 | <0.001 |

Table 9 Normality test of instrumental training accuracy

| items | w_value | p_value |
| --- | --- | --- |
| SFO response | 0.426 | <0.001 |
| MFO response | 0.381 | <0.001 |

### RTs and ACC test

The results indicate that no difference was found on RTs (*t* (57) = -0.65, *p* = .521) and accuracy (*t* (57) = 0.63, *p* = .531) in instrumental training phase.

## Exp 2 test phase RTs and ACC

### Descriptive analysis

Table 10 Descriptive analysis in the test phase

| Response | Cue | RT_mean | RT_sd | ACC_mean | ACC_sd |
| --- | --- | --- | --- | --- | --- |
| SFO response | Neutral cue | 448.796 | 87.933 | 0.981 | 0.033 |
| SFO response | SFO cue | 444.299 | 83.312 | 0.981 | 0.035 |
| SFO response | MFO cue | 461.468 | 97.560 | 0.972 | 0.046 |
| MFO response | Neutral cue | 442.501 | 64.619 | 0.981 | 0.030 |
| MFO response | SFO cue | 444.631 | 66.506 | 0.975 | 0.039 |
| MFO response | MFO cue | 441.015 | 61.427 | 0.980 | 0.030 |

### Normality test

Table 11 Normality test of the test phase RTs

| items | w_value | p_value |
| --- | --- | --- |
| SFO response & Neutral cue | 0.833 | <0.001 |
| SFO response & SFO cue | 0.847 | <0.001 |
| SFO response & MFO cue | 0.807 | <0.001 |
| MFO response & Neutral cue | 0.934 | 0.003 |
| MFO response & SFO cue | 0.929 | 0.002 |
| MFO response & MFO cue | 0.964 | 0.085 |

Table 12 Normality test of test phase accuracy

| items | w_value | p_value |
| --- | --- | --- |
| SFO response & Neutral cue | 0.630 | <0.001 |
| SFO response & SFO cue | 0.613 | <0.001 |
| SFO response & MFO cue | 0.663 | <0.001 |
| MFO response & Neutral cue | 0.636 | <0.001 |
| MFO response & SFO cue | 0.653 | <0.001 |
| MFO response & MFO cue | 0.666 | <0.001 |

**What participants did with the multi-functional snack in Experiment 2**

Twenty-six participants took the multi-functional snack away for other purposes, and thirty-four participants transferred the multi-functional snack for extra 50 euro cents.

1. The DVs used in all analyses have been reciprocally transformed. [↑](#footnote-ref-1)
